# Long noncoding RNAs as potential biomarkers in retinoblastoma: a systematic review and meta-analysis

**DOI:** 10.1186/s12935-020-01281-0

**Published:** 2020-05-29

**Authors:** Jiali Wu, Dashi Qian, Xiaodong Sun

**Affiliations:** 1 Department of Ophthalmology, Shanghai General Hospital (Shanghai First People’s Hospital), Shanghai Jiao Tong University, School of Medicine, Shanghai, 200080 China; 2 Department of Translation and Interpreting, Shanghai Jiao Tong University School of Foreign Language, Shanghai, China; 3 Shanghai Key Laboratory of Fundus Diseases, Shanghai, 200080 China; 4 Shanghai Engineering Center for Visual Science and Photomedicine, Shanghai, 200080 China; 5 National Clinical Research Center for Eye Diseases, Shanghai, 200080 China; 6 Shanghai engineering center for precise diagnosis and treatment of eye diseases, Shanghai, 200080 China

**Keywords:** LncRNA, Retinoblastoma, Meta-analysis, Prognostic

## Abstract

**Background:**

Retinoblastoma is the most common malignant rare intraocular tumor of childhood. Long noncoding RNAs (lncRNAs) have been reported participating in its progression, but their significance remains inconclusive. We conducted this systematic review and meta-analysis to explore specific lncRNA biomarker in patients with retinoblastoma.

**Materials and methods:**

Eligible articles were searched from the Pubmed, Web of Science, Embase and the Cochrane library. Hazard ratios (HRs) and odds ratios (ORs) were extracted or calculated to evaluate the relationship between lncRNAs and retinoblastoma. The meta-analysis part was conducted with STATA v.15 software.

**Results:**

A total of 9 articles with 834 retinoblastoma patients are yielded. Heterogeneity among HRs of overall survival (OS) is notably high (I^2^ = 91.3%, p < 0.001). Subgroup analysis suggests that elevated expression of lncRNA BDNF-AS and MT1JP are favorable factors in OS (pooled HR = 1.89, 95% CI 1.72–2.07, I^2^ = 0%). Six articles included optic nerve invasion as a clinicopathological outcome and showed a notable correlation (pooled HR = 2.38, 95% CI 1.26–3.50, I^2^ = 0.0%). We validate our analysis via the public dataset and also sum up the studies of lncRNA BDNF-AS and MT1JP in other cancers.

**Conclusion:**

Differential expression of lncRNAs has been reported in retinoblastoma. Some of them showed potential in retinoblastoma prognosis and progression.

## Backgrounds

Retinoblastoma is the most common intraocular rare malignant tumor in children [1]. The estimated incidence is approximately 8000 new cases and 3000 deaths in the world annually [2, 3]. Retinoblastoma is not life-threatening as long as being diagnosed early enough and treated appropriately. However, in developing countries, the situation is quite critical, probably due to rather high birth rates and limited medical resources. Approximately 40–70% of patients died in some Asian and African countries [4]. The largest hospital in South Western China used to conduct a retrospective study and found the majority of patients presented at advanced stage [5]. Late diagnosis leads to delayed treatment, which usually means increasing metastasis and decreasing survival rate [6]. Once proliferating into brain through optic nerve, the damage becomes irreparable and fatal. Therefore, effective and early diagnosis is particularly crucial. Exploring novel diagnostic and therapeutic signatures that can be used in retinoblastoma management deserves more study to achieve the aim of eradicating retinoblastoma in the future decade.

Long noncoding RNAs (lncRNAs) are a group of non-coding RNAs that are greater than 200 nucleotides in length. They are widely reported to contribute to tumor progression including proliferation, migration and invasion [7]. Some of them have already been implicated as tumor biomarkers. For example, lncRNA MALAT1 can be applied to predict early and metastatic lung cancers [8]. It is also reported as a cancer suppressor. Professor Ma from famous MD Anderson Cancer Center found it suppressing the metastasis of breast cancer cells [9]. Similarly, emerging studies demonstrate the differential expression of lncRNAs between retinoblastoma and normal tissues, leading us to consider whether they can be potential approaches for early diagnosis and clinical outcomes.

Therefore, we conducted this meta-analysis to evaluate the function of candidate lncRNAs in retinoblastoma in order to find potential prognostic lncRNA markers for further investigation.

## Materials and methods

### Literature search

Two investigators (WJL and QDS) performed this systematic literature search independently through Pubmed, Web of Science, Embase and the Cochrane library (from September 10, 2019 to October 10, 2019). The search terms were used with various conjunctions: (“lncRNA” OR “lincRNA” OR “long noncoding RNA” OR “long untranslated RNA” OR “long intergenic noncoding RNA”) AND (“retinoblastoma”). Related citations of every included article were also manually examined. We only included articles in English language and agreement were achieved on each articles by both of the investigators after careful screening and discussion.

### Selection criteria

Studies from the literature search were included based on the following conditions: (1) LncRNA expression was detected in retinoblastoma tissues; (2) the retinoblastoma tissues were all pathologically diagnosed and classified by ≥ 2 pathologists; (3) the relationship between lncRNA expression and retinoblastoma patient prognosis was investigated, including overall survival and other related clinicopathological parameters; and (4) sufficient data were provided for the hazard ratio (HR) and the 95% confidence interval (95% CI) of survival and clinicopathological parameters. Meanwhile, unsuited studies were excluded if they are: (1) animal studies and reviews; (2) studies based on patient blood and serum; (3) duplicate records; and (4) studies without other sufficient data.

### Data extraction and quality assessment

Two investigators extracted requisite information from each eligible study: first authors, publication year, lncRNA type and expression, sample size, sample type, the HR and 95% CI of lncRNA for survival. Engauge Digitizer v.10.11 software and Tierney’s spreadsheet were utilized to obtain the HR when it was not offered directly. HR from multivariate analysis was preferred over that from the univariate analysis as it accounted for confounding factors. Quality assessment of each eligible study was performed based on the acknowledged Newcastle–Ottawa Quality Assessment Scale (NOS) with scores ranging from zero to nine. A study with a NOS score higher than 6 is regarded as high quality.

### Data processing

Datasets of GSE125903 were normalized before calculation. Differential expressing genes (DEGs) were analyzed by R packages of “limma” (http://www.bioconductor.org/packages/release/bioc/html/limma.html). Limma is an open source package on Bioconductor platform. We use Benjamini–Hochberg method to get the adjusted p values, lmfit and eBayes functions to get the different expressed genes. The cutoff of adjusted p values was set to 0.05 and the cutoff of |log2FC| was 1.5. The genes which meet the two conditions above were identified as DEGs. We use ggpubr and ggthemes to draw volcano plots for each datasets, with an x axis of “log2(Fold Change)” and an y axis of “−log10(Adjust p*-*value)”. The heatmap was done by R package of “pheatmap”.

### Statistical analysis

As different lncRNAs act differently in retinoblastoma, pooling HRs directly would lead to great heterogeneity, we assessed the heterogeneity among studies and conducted the subgroup analysis of HRs based on different lncRNAs. Heterogeneity of the results was estimated by the Q test and I^2^ statistics. We selected the random pooling model, to be on the safe side. HR > 1 suggested a significant association of lncRNA overexpression with poor survival, and HR < 1 indicated that high lncRNA expression predicted long survival. All these calculations were completed with STATA v.15 software.

## Results

### Characteristics of the included studies

**Fig. 1:**
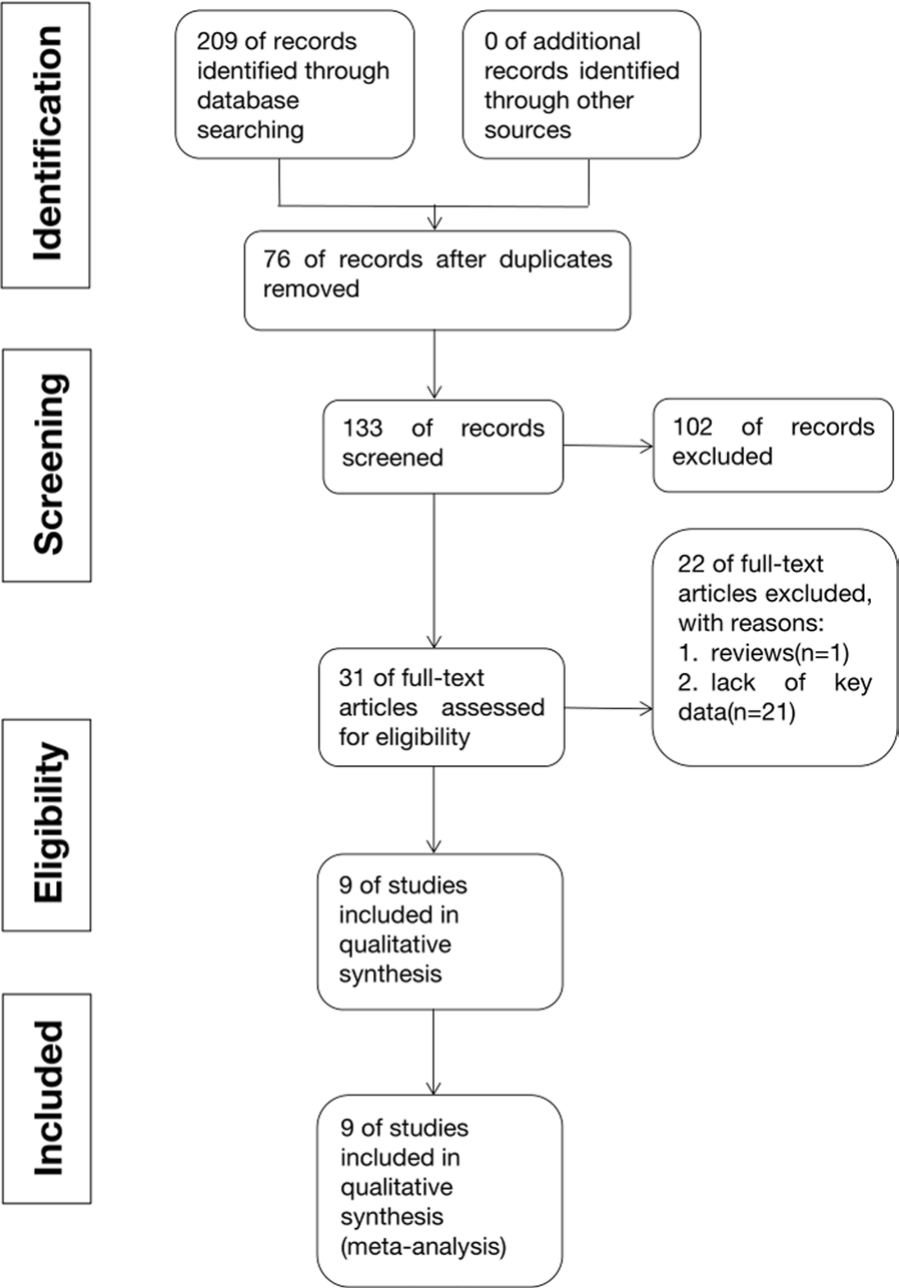
Flow diagram of this systematic review and meta-analysis

As shown in Fig. 1, a total of 209 articles were identified from online resources. Among them, 9 articles with 834 retinoblastoma patients published between 2015 and 2019 in China were included in our analysis. The relevant lncRNAs include PVT1 [10], AFAP1-AS1 [11], HOTAIR [12], BANCR [13], H19 [14], DANCR [15], LINC00202 [16], MT1JP [17] and BDNF-AS [18]. 7 of them used OS as a main outcome and 2 of them used both overall survival (OS) and disease-free survival (DFS) for survival analysis. OS means the percentage of patients still alive at the endpoint while DFS means the percentage of patients without any symptoms of the retinoblastoma. The expression was detected by quantitative polymerase chain reaction (qPCR) in human retinoblastoma tissue specimens. More than half of the studies included applied median value as the cut-off value to stratify patients into high of low expression group. 6 of the articles reported the HRs directly while the remaining used survival curves. Most included articles are of high quality with a rather high NOS score. More details are shown in Table 1.

**Table 1:** Characteristics of the included studies

First author	Year	LncRNA	Expression	Sample size	Cut-off value	PT	Survival	Survival analysis	HR availability	NOS score
Xue-Zhi Wu	2019	PVT1	Upregulated	78 retinoblastoma tissue specimens 30 normal retina samples	Median	No	OS	U	Kaplan–Meier	8
Fengqin Hao	2018	AFAP1-AS1	Upregulated	58 retinoblastoma tissue specimens 10 non-cancerous retina tissue specimens	Median	No	OS	U, M	Kaplan–Meier	8
Ge Yang	2018	HOTAIR	Upregulated	350 eyes were collected from 276 patients	NA	No	OS	U, M	Kaplan–Meier	5
Shizheng Su	2015	BANCR	Upregulated	60 retinoblastoma samples 4 normal retina samples	Median	NA	OS	U, M	Kaplan–Meier	7
Li Li	2016	H19	Upregulated	80 retinoblastoma tissue samples	NA	No	OS	U, M	NA	5
Jingxian Wang	2018	DANCR	Upregulated	57 cases of RB patients and matched health controls	NA	NA	OS, DFS	U	Kaplan–Meier	8
Guigang Yan	2019	LINC00202	Upregulated	50 cases of RB tissues and paired normal tissues	Fold change	NA	OS, DFS	U	Kaplan–Meier	8
L.-L. Bi	2018	MT1JP	Downregulated	44 retinoblastoma tissues and matched non-tumor tissues	Median	No	OS	M	Kaplan–Meier	8
Weiwei Shang	2018	BDNF-AS	Downregulated	131 patients with RB 35 normal retinal specimens	Median	NA	OS	U, M	Kaplan–Meier	8

U: univariate analysis; M: multivariate analysis; PT: preoperative treatment; K–M curve: Kaplan–Meier curve; NA: not available

### The relationship between lncRNAs and patient survival

**Fig. 2:**
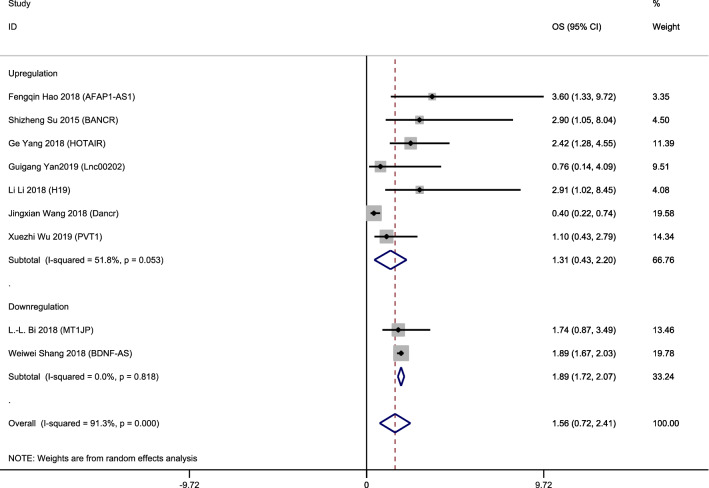
Subgroup analysis of OS by lncRNA expression in retinoblastoma

**Fig. 3:**
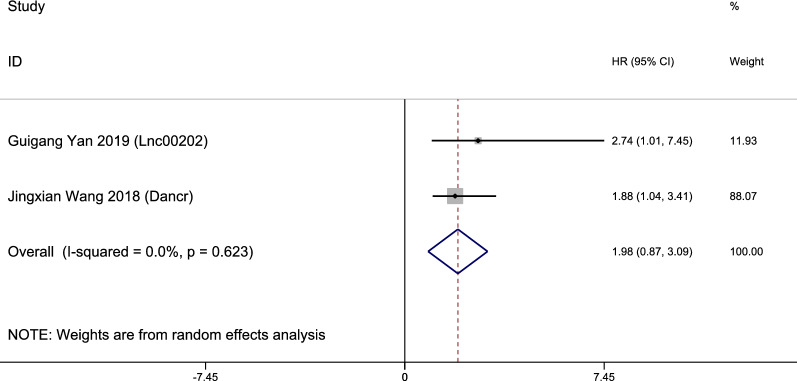
Forest plot of HRs of high lncRNA expression and DFS

LncRNAs PVT1, AFAP10AS1, HOTAIR, BANCR, H19, DANCR and LINC00202 were up-regulated in retinoblastoma tissues while MT1JP and BDNF-AS were down-regulated. Owing to the rather significant heterogeneity (I^2^ = 91.3%, p<0.001), a random model was employed to calculate the 95% CI and pooled HR. To decrease the heterogeneity, we performed subgroup analysis. It suggested no significance between overall high expression and poor OS (pooled HR = 1.31, 95% CI 0.43–2.20, I^2^ = 51.8%; Fig. 2). Individually, high expression of lncRNA AFAP1-AS1 (HR = 3.6, 95% CI 1.33–9.72), BANCR (HR = 2.9, 95% CI 1.05–8.04) and H19 (HR = 2.91, 95% CI 1.02–8.45) are obviously associated with worse OS. In contrast, increased levels of LncRNA BDNF-AS and MT1JP were favorable factors in OS (pooled HR = 1.89, 95% CI 1.72–2.07, I^2^ = 0%; Fig. 2). Both LncRNA BDNF-AS and MT1JP have been reported decreased in quite a few cancers including gastric cancer, bladder cancer and lung cancer, suggesting their potential role as a rather broad and sensitive tumor suppresser. Two of the articles included provided DFS data and we also found no significance between their overall high expression and poor DFS (pooled HR = 1.98, 95% CI 0.87–3.09, I^2^ = 0.0%; Fig. 3).

### The relationship between lncRNAs and clinicopathological outcomes

**Fig. 4:**
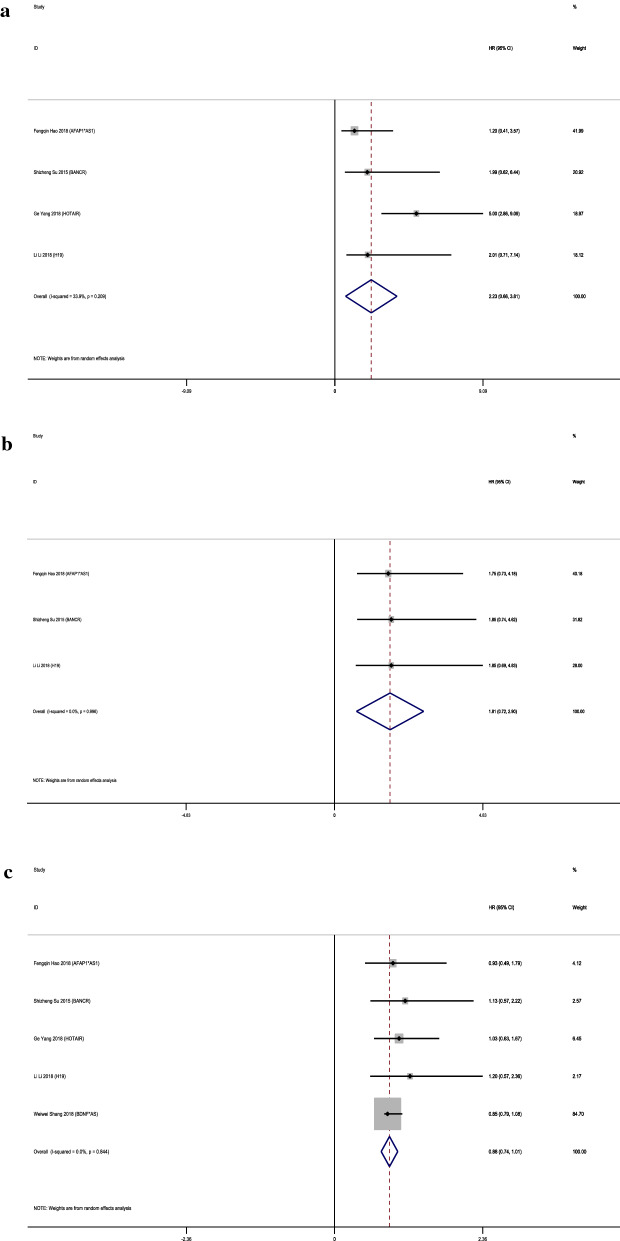

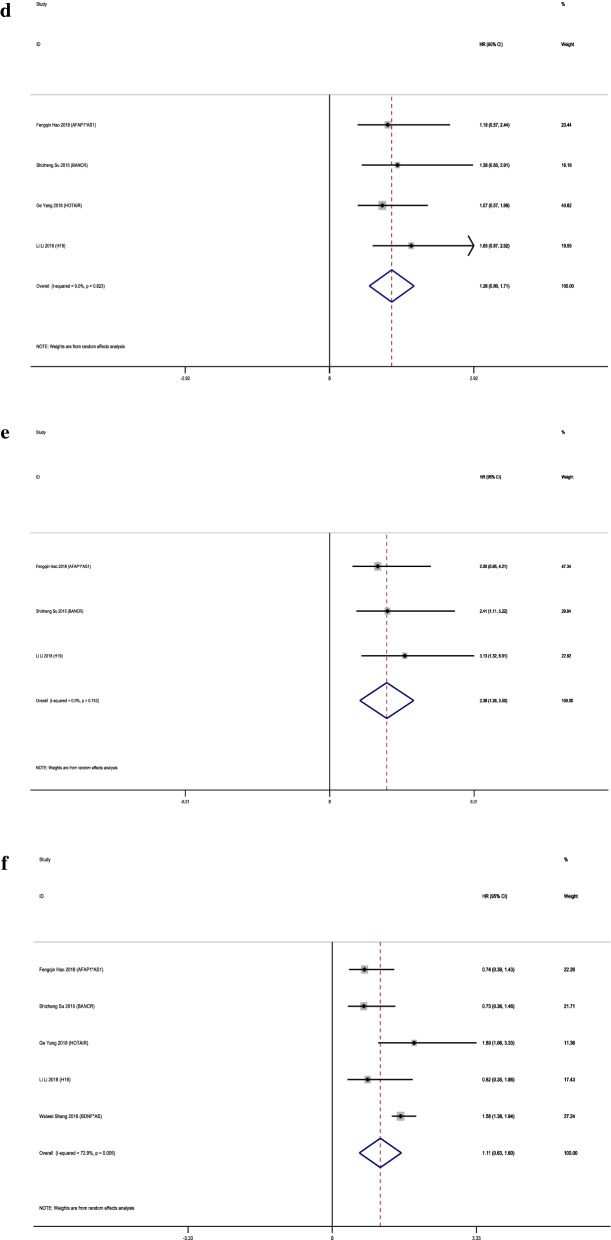
Forest plots of the association of lncRNA expression with clinicopathological parameters: **a** tumor size; **b** choroidal invasion; **c** gender; **d** laterality; **e** optic nerve invasion and **f** pathologic grade

**Fig. 5:**
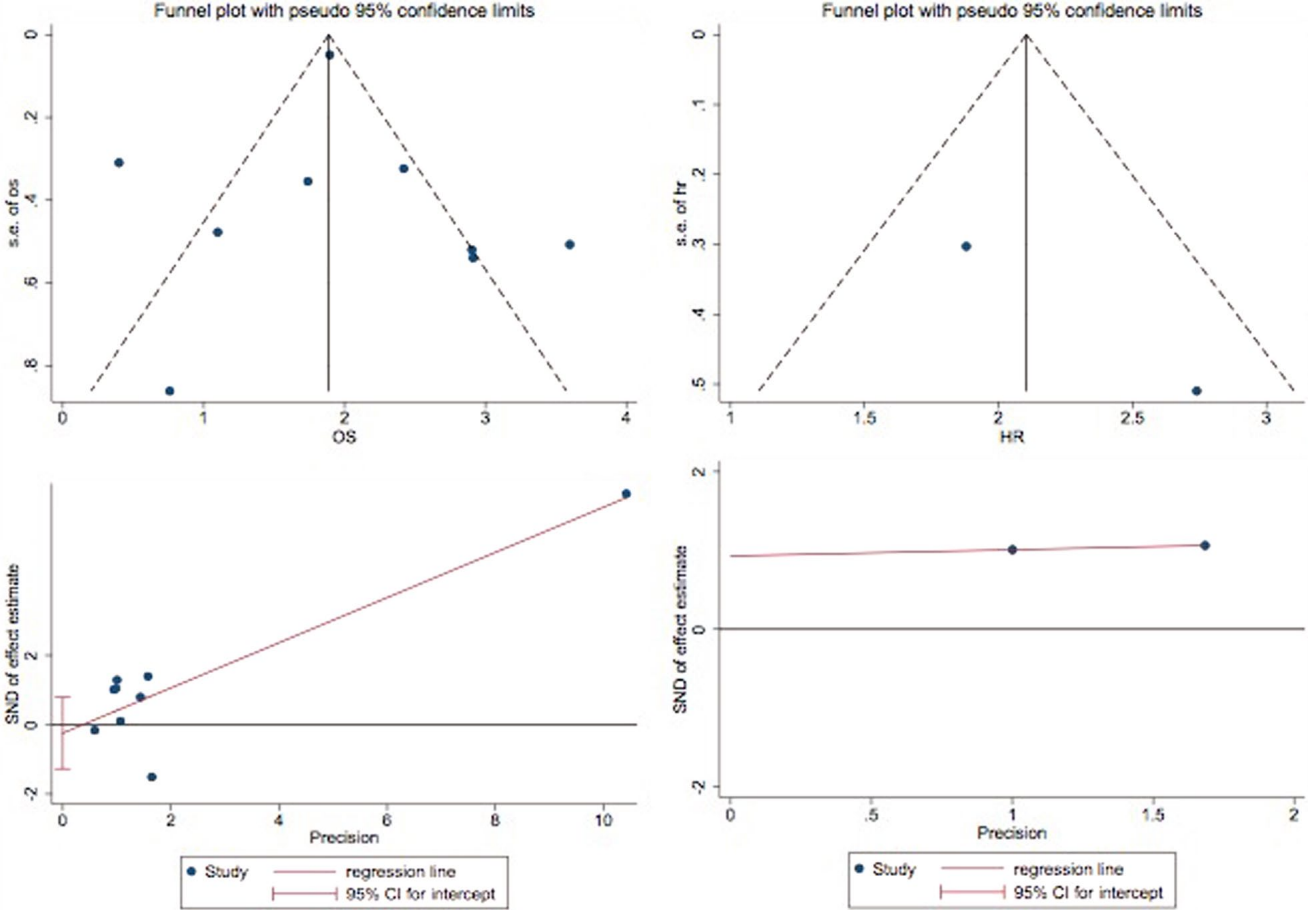
Tests for publication bias of OS

The clinicopathological outcomes mentioned by these 9 articles include tumor size, choroidal invasion, gender, laterality, optic nerve invasion and pathologic grade. All lncRNAs had no remarkable relationship with tumor size, choroidal invasion, gender, laterality and pathologic grade (tumor size: p = 0.209, choroidal invasion: p = 0.996, gender: p = 0.844, laterality: p = 0.823 and pathologic grade: p = 0.005). LncRNA HOTAIR was significantly correlated with larger tumor size (HR = 5, 95% CI 2.86–9.09). Besides, it also related with TNM classification (HR = 1.89%, 95% CI 1.06–3.33). 6 articles included applied optic nerve invasion as a clinicopathological outcome and showed a notable correlation (pooled HR = 2.38, 95% CI 1.26–3.50, I^2^ = 0.0%; Fig. 4). Optic nerve invasion is known as a major risk factor for retinoblastoma mortality [19]. There is a relation between the extent of invasion and metastasis. Once the invasion come to the cut end of the optic nerve, the mortality increases to 50 ~ 80% [20]. However, it remains unsolved how to predict the invasion before it happens or evaluate the involvement during development [21]. Probably these three lncRNAs can provide some new hints. More details are shown in Fig. 5.

### Publication bias and sensitivity analysis

**Fig. 6:**
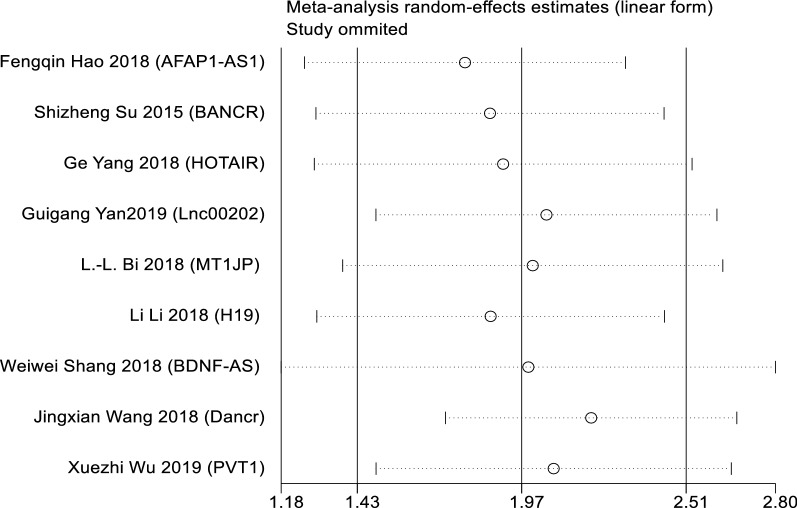
Sensitivity analysis of studies on OS

Egger’s publication bias plot and Begg’s funnel plot were constructed to explore the potential publication bias in this study. Two plots did not display apparent asymmetry (Egger’s test: p = 0.749 and Begg’s test: p = 0.553), which showed no significant publication bias (Fig. 5). In addition, the sensitivity analysis suggested the robustness of the results because eliminating any study did not change the results significantly (Fig. 6).

### Action mechanisms of lncRNAs in retinoblastom

Furthermore, we concentrated on potential targets and pathways of these lncRNAs included in our study (Table 2). Most of them sponge with microRNA to influence retinoblastoma progression.

**Table 2:** The potential targets and function of the lncRNAs included in our study

First author	LncRNA type	Expression	Potential target(s)	Pathways
Xuezhi Wu	PVT1	Upregulated	miR-488-3p	↑ Cancer progression; ↓ cell apoptosis; ↓ G1/S arrest; ↑ Migration and invasion
Fengqin Hao	AFAP1-AS1	Upregulated	NA	↑ Cell proliferation; ↑ cell cycle progression; ↑ cell migration and invasion
Ge Yang	HOTAIR	Upregulated	miR‐613	↓ Cell apoptosis; ↑ Cell proliferation and cell viability
Shizheng Su	BANCR	Upregulated	NA	↑ Cell proliferation, migration and invasion; ↑ Retinoblastoma progression
Li Li	H19	Upregulated	NA	↑ Cell proliferation, apoptosis; migration and invasion; ↑ Retinoblastoma progression
Jingxian Wang	DANCR	Upregulated	miR-34c; miR-613	↑ Proliferation, migration, invasion, and epithelial-mesenchymal transition (EMT); ↑ tumor growth
Guigang Yan	LINC00202	Upregulated	miR-3619-5p	↑ Cell proliferation, migration and invasion
L.-L. Bi	MT1JP	Downregulated	Wnt/β-Catenin	↑ G0/G1; ↑ cell apoptosis; ↓ Cell proliferation, migration and invasion
Weiwei Shang	BDNF-AS	Downregulaed	NA	↓ Cancer proliferation and migration; ↑ Cell-cycle arrest

### Validation of the candidate lncRNAs

**Fig. 7:**
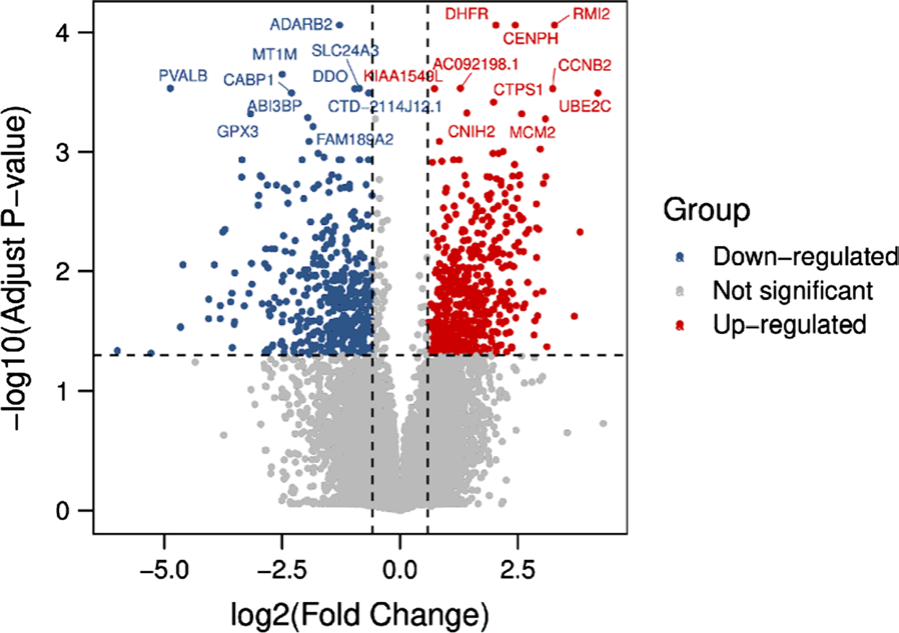
Volcano plot of GSE125903

**Fig. 8:**
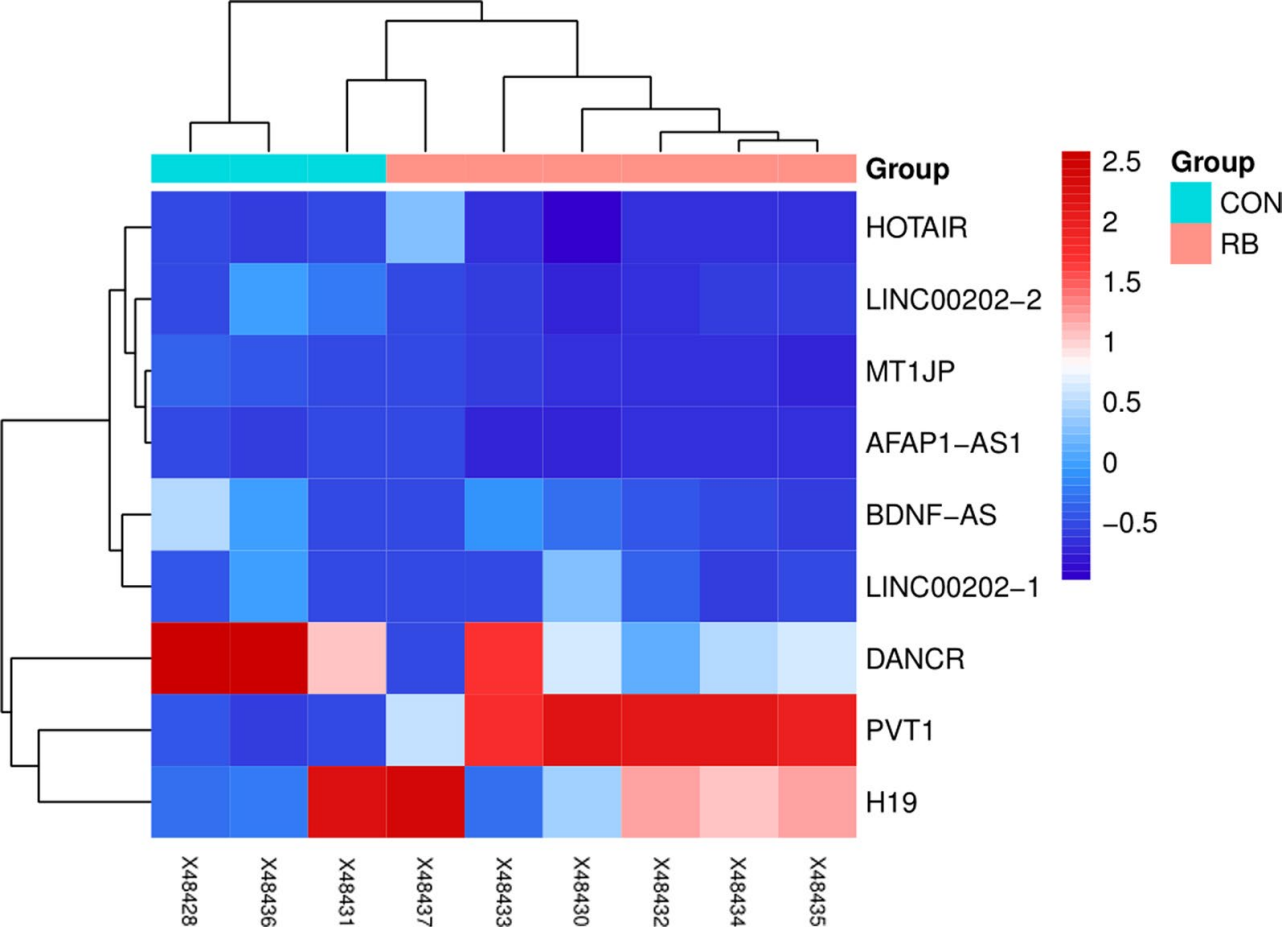
Heatmap of 9 lncRNAs in GSE125903

Right now, we have figured out two potential lncRNAs. For the further validation, we searched the public datasets and found GSE125903 (Fig. 7). It was the first transcriptomic report on pediatric retinoblastoma tumor based on 7 retinoblastoma tissue and 3 control retina tissue [22]. We downloaded its original data and analyzed the 9 lncRNAs discussed in our analysis. The results were showed in a heatmap (Fig. 8). LncRNA MT1JP and BDNF-AS were slightly decreased in retinoblastoma tissues, which was consistent with our analysis. In addition, lncRNA DANCR, PVT1 and H19 also showed significant changes, which deserved more attention.

## Discussion

Retinoblastoma accounts for 3% of all childhood cancers and is one of the most malignant intraocular tumors [23]. Despite of the development of overall health care, the mortality of retinoblastoma still remains relatively high in Asia, especially in China, partly due to the likely delayed diagnosis [24]. Thus, looking for suitable and sensitive screening biomarkers would benefit clinicians a lot in taking actions in time.

In the past few years, various lncRNAs have been reported in the pathogenesis and progression of retinoblastoma. Last year, Pluousiou et al. did a comprehensive review of lncRNAs involved in retinoblastoma [25], however, right now, no study has described the relation between lncRNAs and patient survival or clinicopathological outcomes systematically.

In our study, we analyzed the role of 9 lncRNAs in retinoblastoma survival and clinical indicators. Seven upregulated lncRNAs showed no overall remarkable relationship with OS. Two downregulated lncRNAs: BDNF-AS and MT1JP predicted favorable OS. We further searched the public dataset to validate our analysis. However, we also found both of them have been reported in quite a few cancers (Table 3), thus their specificity for retinoblastoma needs further verification. High expression of Lnc00202 and BANCR both indicated poorer DFS while taken together, this result is not significant.

**Table 3:** Studies including lncRNA BDNF-AS and MT1JP in cancers

First author	Year	Diseases	Sample size	Assay	Expression	Potential targets
LncRNA BDNF-AS						
Huaying Zhao [26]	2018	Oesophageal cancer	45 pairs of surgical primary EC tissues	qPCR	Downregulated	miR‐214
Huimin Zhang [27]	2018	Cervical cancer	125 pairs of cancer cervix tissues	qPCR	Downregulated	BDNF
Hui Zhi [28]	2019	Colorectal cancer (CRC)	20 pairs of CRC tissues	qPCR	Downregulated	GSK-3β
Wensheng Li [29]	2018	Prostate cancer (CaP)	141 pairs of surgical CaP tissues	qPCR	Downregulated	NA
Qiang Huang [30]	2018	Osteosarcoma (OS)	114 OS samples 35 paired non-cancerous samples	qPCR	Downregulated	cleaved caspase-3
Farbod Esfandi [30]	2019	Gastric cancer	30 pairs of cancer specimens	qPCR	Downregulated	NA
LncRNA MT1JP						
Ying Xu [31]	2018	Gastric cancer	99 pairs of GC tissues and neighboring noncancerous tissues	qPCR	Downregulated	MT1JP/MiR-214-3p/RUNX3
Gang Zhang [32]	2018	Gastric cancer	5 pairs of normal and GC tissues + 75 paired tissues	qPCR	Downregulated	MT1JP/MiR-92a-3p/FBXW7
Donglei Zhu [33]	2019	Breast cancer	56 paired samples of breast cancer tissue and adjacent normal breast tissues	qPCR	Downregulated	miR-24-3p
Haifeng Yu [34]	2019	Bladder tumor	35 paired samples of bladder cancer samples and adjacent no‐tumor samples	qPCR	Downregulated	miR-214-3p
Jiyong Ma [35]	2019	Lung cancer	30 non-small cell lung cancer (NSCLC) and adjacent normal tissues	qPCR	Downregulated	miRNA-423-3p/Bim

Among the 5 main clinicopathological parameters, lncRNAs had significant relation with optic nerve invasion. Optic nerve invasion is one of the most significant risk factor for eye enucleation and mortality and with the increase of involvement, the mortality risk increases, too. Predicting or managing the development of nerve invasion remains a demanding task [36] while lncRNAs might be somewhat of benefit.

We also summed up the mechanisms of lncRNAs in retinoblastoma, which might be helpful for further comprehensive recognition.

Limitations of our meta-analysis include: (1) All of the studies included are conducted in China. We propose studies outside China to compare and confirm our results. (2) Sample size of most studies included are rather limited and larger researches are still needed. (3) Some of the studies did not provide HRs directly and we collected the necessary information from survival curves, which might be not so accurate. (4) We only collected the targets and pathways of lncRNAs studied in the articles we included. More regulatory mechanisms are available in articles we did not enrolled.

## Conclusions

Our study systematically reviewed and analyzed the abnormally expressed lncRNAs in retinoblastoma. Based on all eligible evidence, we found two lncRNAs:BDNF-AS and MT1JP as potential prognostic biomarker for retinoblastoma. Considering our existed limitations and further needs, larger-scale prospective investigations are warranted for validation.

## Data Availability

All data are included in this article.
